# Resonant photoacoustic spectrometer enhanced by multipass absorption for detecting atmospheric CH_4_ at the ppb-level

**DOI:** 10.3389/fchem.2022.1021145

**Published:** 2022-09-21

**Authors:** Qiang Liu, Yi Sun, Xuanbing Qiu, Guqing Guo, Lin Li, Ting Gong, Chuanliang Li

**Affiliations:** ^1^ Key Laboratory of Atmospheric Optics, Anhui Institute of Optics and Fine Mechanics, HFIPS, Chinese Academy of Sciences, Hefei, China; ^2^ Shanxi Engineering Research Center of Precision Measurement and Online Detection Equipment and School of Applied Science, Taiyuan University of Science and Technology, Taiyuan, China

**Keywords:** photoacoustic spectroscopy (PA), herriott-type, multipass enhancement, wavelength modulation spectroscopy (laser spectroscopy), atmospheric CH4 detection

## Abstract

A resonant photoacoustic spectrometer (PAS) was developed for detecting trace atmospheric CH_4_. The sensitivity of the PAS was significantly increased *via* a Herriott-type multipass cell with a beam pattern concentrated in the cavity. The effective optical pathlength of the PAS can be optimized to 6.8 m with 34 reflections and a diameter of 6 mm. A distributed feedback diode laser at 1,653 nm was employed as the light source, and wavelength modulation spectroscopy was used for the 2nd harmonic signal to reduce the noise of the system. The resonant cell of PA and optimal modulation frequency were obtained by varying the measurements. In comparison with a single path, the sensitivity of the multipass strategy was improved 13 times. To evaluate the long-term stability and minimum detection limit (MDL) of the system, an Allan variance analysis was performed, and the analysis illustrated that the MDL accomplished 116 ppb at an average time of 84 s. The system was utilized for 2 days test campaign to validate the feasibility and robustness of the sensor. The system provides a promising technique for online monitoring of greenhouse gasses.

## Introduction

Detecting trace gas is vital in processes that monitor atmospheric environment, such as air quality inspections, pollutant emission detections and greenhouse gas measurements (Bogue, 1981; Werle et al., 1998). Methane (CH_4_) is a flammable and explosive gas that considerably influences the greenhouse effect of the atmosphere (Wuebbled and Hayhoe, 2002; Bamberger et al., 2014; Nisbet et al., 2014; Gong et al., 2021). Therefore, real-time, rapid and accurate detection of CH_4_ concentrations is crucial for environmental monitoring and physical and chemical atmospheric research. Photoacoustic spectroscopy (PAS) is superior to other trace gas detection methods due to its fast response, high selectivity and sensitivity (Harren et al., 2000; Gong et al., 2017; Wu et al., 2017; Yin et al., 2017; Gong et al., 2018; Chen et al., 2020).

PAS relies on the PA effect that is generated by the absorption of light by the sample (Bell, 1880). Therefore, the PA signals could be enhanced under a higher output power of the light source (Kosterev et al., 2008; Wang et al., 2011; Wang and Wang, 2014; Gong et al., 2019; Yin et al., 2021). In recent years, much effort has been made to improve the detection sensitivity of PAS. Several groups have reported mid-infrared PA sensors that exhibit high sensitivity based on quantum cascade lasers, which correspond to molecular strong fundamental bands and have higher output power compared to that of the near infrared region (Ma et al., 2013; Peltola et al., 2013; Krzempek et al., 2018; Wu et al., 2019). However, mid-infrared lasers are still limited to practical applications due to their cost. Therefore, it is a better choice to increase the absorption optical path in the near-infrared in consideration of cost. Qiao et al. (2021) constructed a multipass quartz-enhanced PA sensor that allowed the laser beam to pass through the quartz tuning fork (QTF) prong spacing six times. The signal was enhanced approximately 3.2 times compared to that of the single-pass structure. The QTF widely serves as a resonant microphone in PA sensors because of its high quality factor, but the QTF is susceptible to disturbance by water vapour in atmospheric monitoring (Shanwen et al., 2012). Zhang et al. developed a multipass PA gas sensor utilizing a nonresonant PA cell with 29 reflections, in which the minimum detection limit can reach 12.2 ppb (Zhang et al., 2020). The researchers replaced the windows of the PA cell with concave mirrors, forming a Herriott-type cell. However, the fixed distance of cell mirrors limits the number of reflections and the mirrors are easily contaminated in atmospheric detection.

In this work, we developed a low-cost and high-sensitivity multipass enhancement PA sensor based on resonant PAS for gas atmospheric CH_4_ detection. The detection sensitivity was improved 13 times by a Herriott-type resonant PA cell with 34 reflections. The total absorption length was 6.8 m. In addition, the experimental parameters of the sensor were optimized by 200 ppm CH_4_ standard gas. The minimum detection limit (MDL) of this sensor can reach 116 ppb at an 84 s average time. To validate the stability of the system, the PA sensor was deployed to measure atmospheric CH_4_ for 2 days.

## Design of the photoacoustic spectrometer sensor

### Design of the Herriott-type multipass PA cell

**FIGURE 1:**
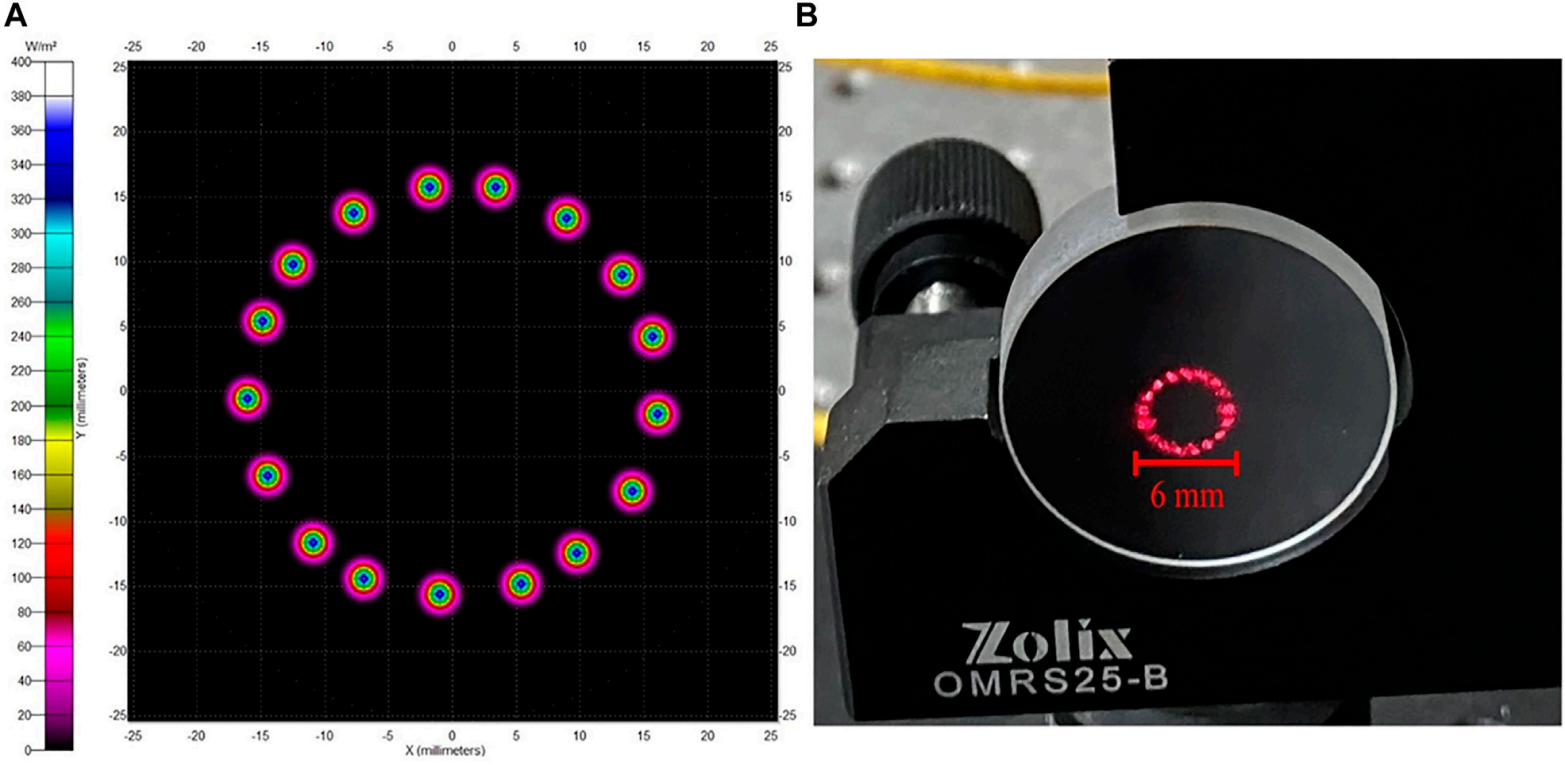
**(A)** Simulation result of the beam pattern; **(B)** Observed footprint of laser beam at 640 nm.

Based on the ABCD matrix, the Herriott beam pattern was derived as illustrated in Figure 1A. The size of the beam pattern and the number of reflections can be adjusted by changing the distance of the mirrors and the laser incident angle. An optimum of 34 reflections was chosen in consideration of the volume of the PA cell, so it is reflected 17 times for each mirror. Two windows are used to enclose the PA cell, and cavity mirrors are placed outside to prevent the sample gas from being comtaminated. Two mirrors with a reflectivity of −95% and 30.48 cm curve radius are separated by 28.5 cm. As presented in Figure 1B, the diameter of the beam footprint on the mirror is approximately 6 mm, which can pass through an acoustic resonator with a diameter of 9 mm. The cell windows are coated with anti-reflection film at both 640 and 1,653 nm, which correspond to indication and signal lasers, respectively. The anti-reflective coating in both spectral regions is better than 1% within ± 10 nm bandwidth.

### Experimental setup and its optimization

**FIGURE 2:**
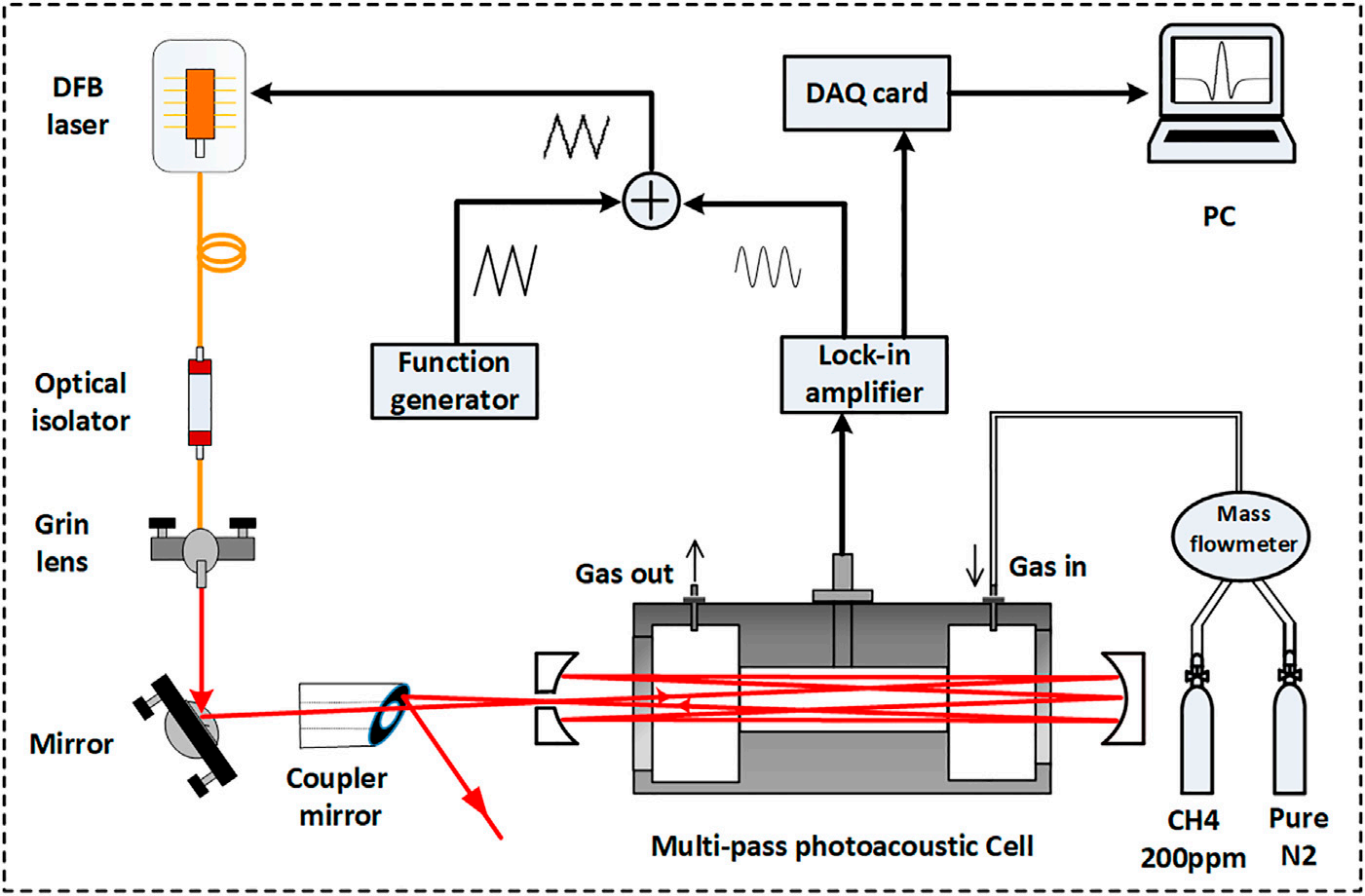
Schematic experimental setup of a multi-pass PA sensor for trace gas detection.

Figure 2 shows the schematic structure of the multipass PA sensor for the gas detection system. A distributed feedback diode (DFB) laser operating at 1,653 nm with an output power up to 15 mW is used as an excitation source. The absorption line is free of interference from atmospheric gases in this band (Iseki et al., 2000). To obtain higher output power, the laser temperature and current are set to 27.7°C and 105.42 mA, respectively, in the experiment. An optical isolator is connected to the laser tail fiber to prohibit the feedback of the partially reflected laser beam from influencing the DFB laser. The output of the laser passes through a grin lens, is reflected by a plane mirror, and is finally coupled into the Herriott-type cell through a coupled mirror. Analogous to wavelength modulation spectroscopy, a triangle wave of 2 Hz generated by the function generator and the sinusoidal wave provided by a lock-in amplifier (Stanford Research Systems, Model SR830) are superimposed through an adder to drive the injection current of the DFB laser. The signal from the microphone (BSWA, MA221) is magnified 1,000 times by a built-in amplifier and demodulated by the lock-in amplifier at the 2^nd^ harmonic (2*f*). Ultimately, the demodulated signal is recorded by the DAQ (National Instruments, PCIe-6353) and stored by a computer. The range of the flow rate of the homemade mass flowmeter was 100–300 ml/min. The concentration of the sample gas was calibrated by a series of standard mixing gases of CH_4_ diluted by nitrogen (N_2_). The pressure of the PA cell was controlled to be −100 Torr and the setup was worked in room temperature 26°C.

**FIGURE 3:**
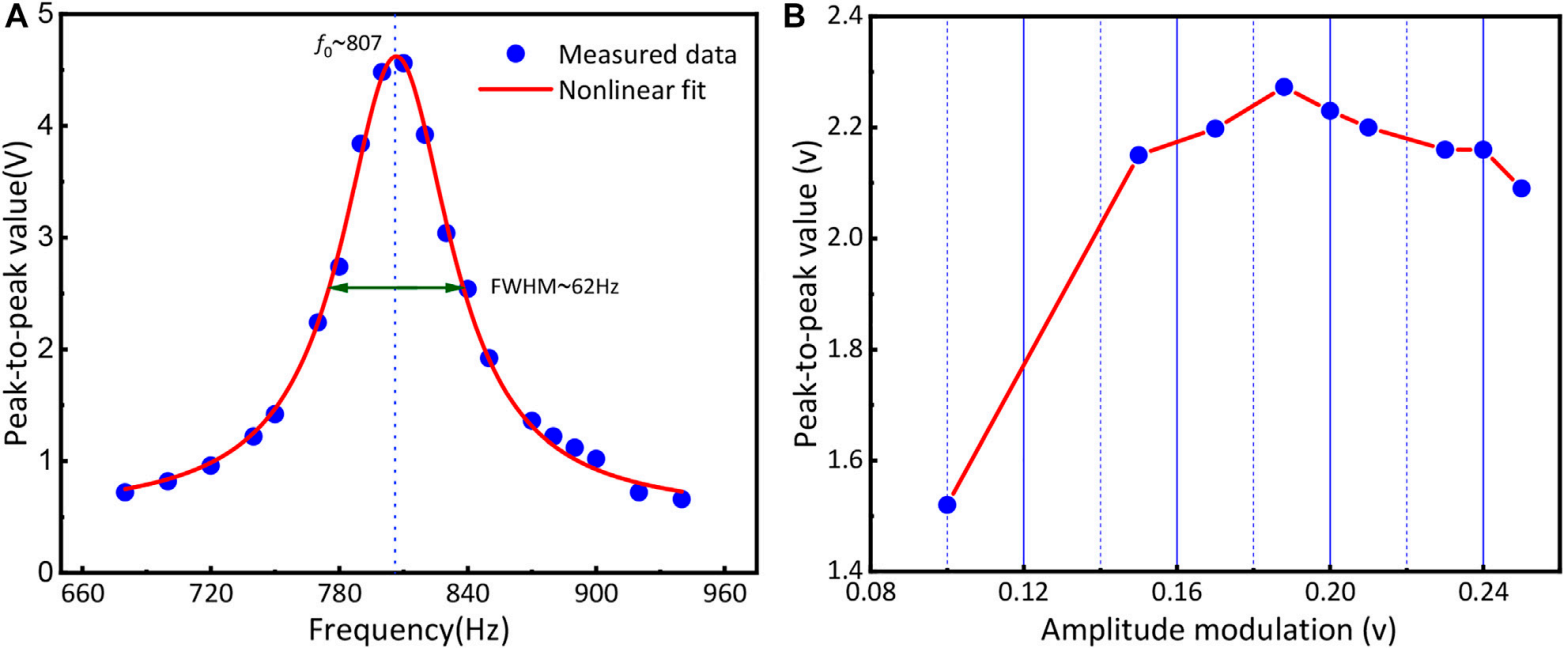
**(A)** Frequency response (*f*_*0*_) of the PA cell; **(B)** Peak value of standard gas at different amplitude modulation.

Modulation frequency and modulation amplitude are two crucial parameters with respect to the amplitude of PA. The optimum performance can be derived when the modulation frequency matches the resonant frequency (*f*_*0*_) of the PA cell. The resonance frequency was obtained by using a high concentration of CH_4_ gas. As depicted in Figure 3A. The maximum amplitude appears at *f*_*0*_ = 807 Hz and corresponds to half of the resonance of the PA cell (Ma et al., 2019), which is associated with a quality factor of 13. Hence, the *f*_*0*_ of the PA cell is 1,614 Hz. The relationship between the PA signal peak and modulation amplitude was measured under 200 ppm standard CH_4_ gas. During the experiment, the relationship between the PA signals and the amplitude of the modulated sinusoidal wave is shown in Figure 3B. The PA signal is strongest at a modulation amplitude of 0.188 V. In the experiment, the sensitivity and time constant of the lock-in amplifier were set at 10 μV and 3 ms, respectively. Under the optimal condition of detection, the performance of the multipass enhancement resonant PA sensor was investigated using the setup depicted in Figure 2.

**FIGURE 4:**
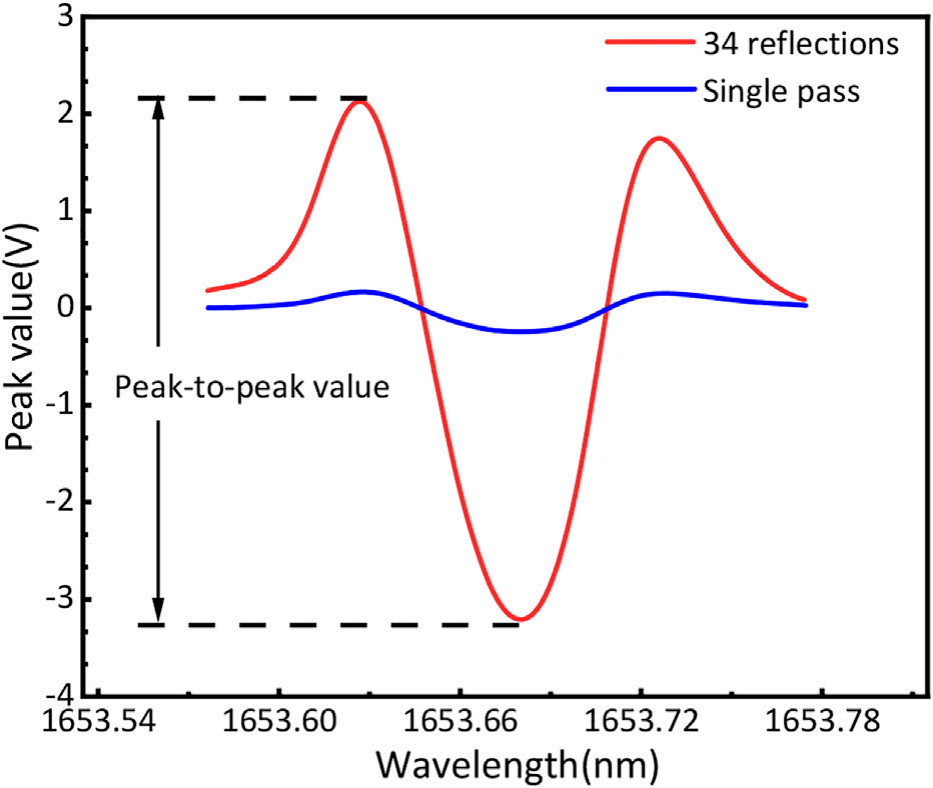
2*f* signal of the 34 reflected PA system (blue line) and single-pass conventional standard PA system (red line), respectively.

The comparison of the 2*f* signal of the multipass system and single-pass configuration is shown in Figure 4. The peak-to-peak value for the multi-pass PA sensor was estimated at 5.2 V in comparison to the 0.4 V obtained *via* the single-pass configuration. For the multipass PA sensor, the integral laser power in the PA cell can be calculated according to the following equation:

$$P=\overset{n}{\underset{k=0}{\sum }}\left(P_{0}\cdot R^{k}\right)$$ (1)

Where *P*_*0*_ is the output power of the DFB laser, *R* is the reflectivity of the cavity mirrors and is of about 95%, *n* is the reflection times of the laser and is of 34. Thus *P* is calculated to be −16.6 times of *P*_*0*_. The gain of the photoacoustic signal is reduced due to the loss of the window mirror and the difference of optical-acoustic pattern matching between single-pass and multipass.

## Results and discussion

**FIGURE 5:**
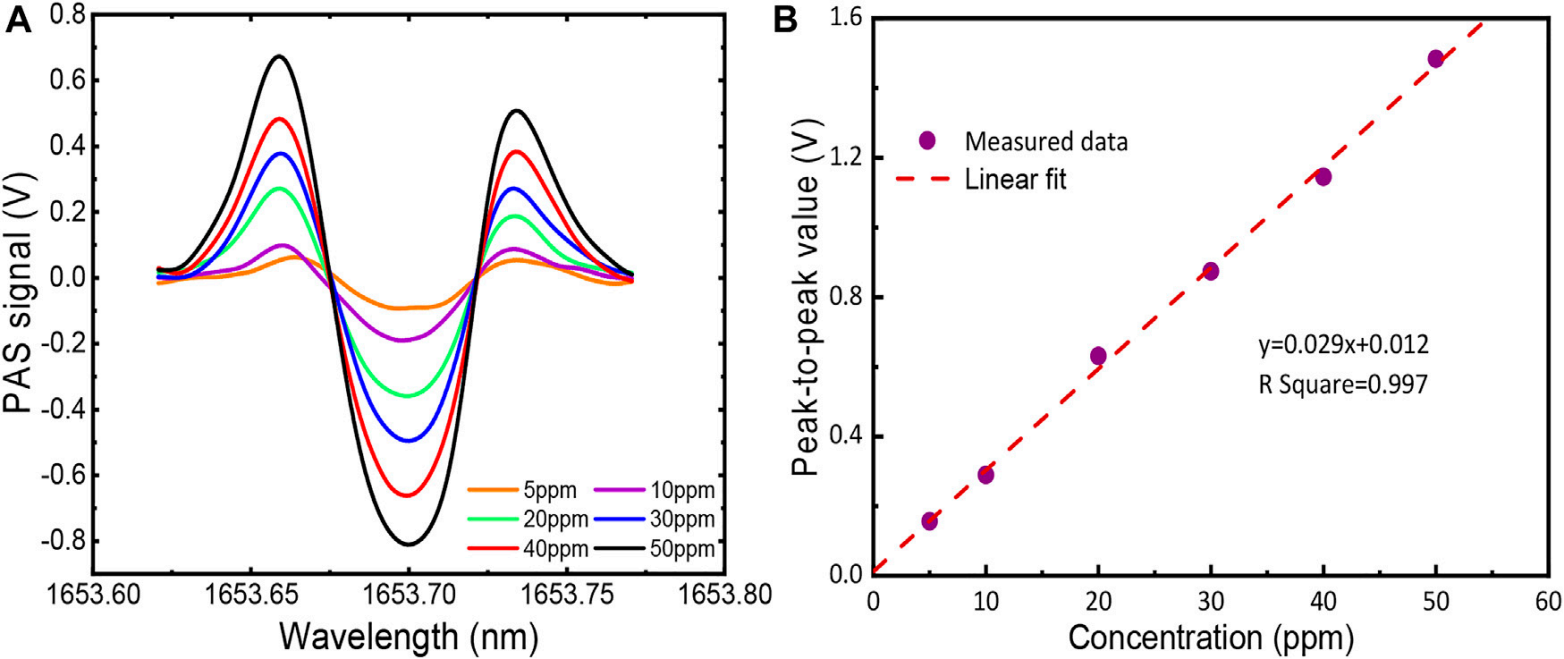
**(A)** Observed 2*f* signals of CH_4_ at different concentrations; **(B)** Linear fitting of amplitudes of PA signals at different CH_4_ concentrations.

**FIGURE 6:**
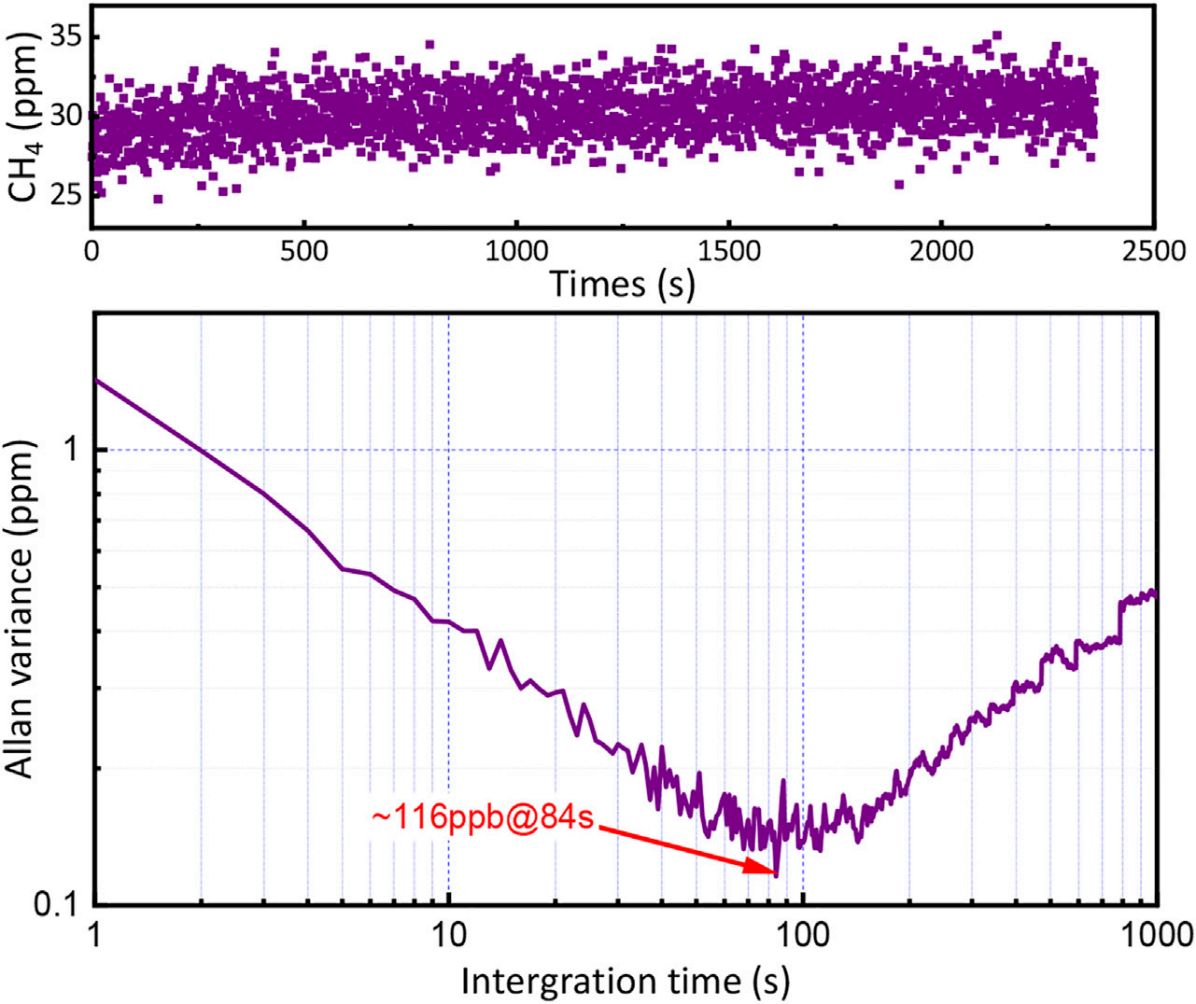
Row data of individual concentration measurements over 40 min (top) and Allan variance as a function of integration time (bottom).

To further verify the linear characteristics of the multipass PA gas sensor, several concentrations of CH_4_ were filled into the PA cell, which was controlled by the mass flowmeter. A series of concentrations (5, 10, 20, 30, 40, and 50 ppm) of CH_4_/N_2_ gas were obtained by diluting the 200 ppm CH_4_ with pure N_2_. The spectral lines of CH_4_ at different concentrations are presented in Figure 5A. Then the relationship of the signal amplitude of the spectra with concentrations was obtained by linear fitting, as shown in Figure 5B. The R square equal to 0.993 indicates that the multipass PA gas sensor exhibits a good linear characteristic for CH_4_ gas detection. In addition, the linear function can be easily used to calculate any unknown CH_4_ concentration. The signal of 30 ppm CH_4_ was measured for 1 h to assess the detection limitation and stability of the sensor. A total of −2,400 data points were obtained with a scan frequency of 1 Hz. As shown in Figure 6 (bottom), the Allan variance is a function of the measurement time. The figure indicates that the minimum detection limit appears at 84 s and approaches 116 ppb.

**FIGURE 7:**
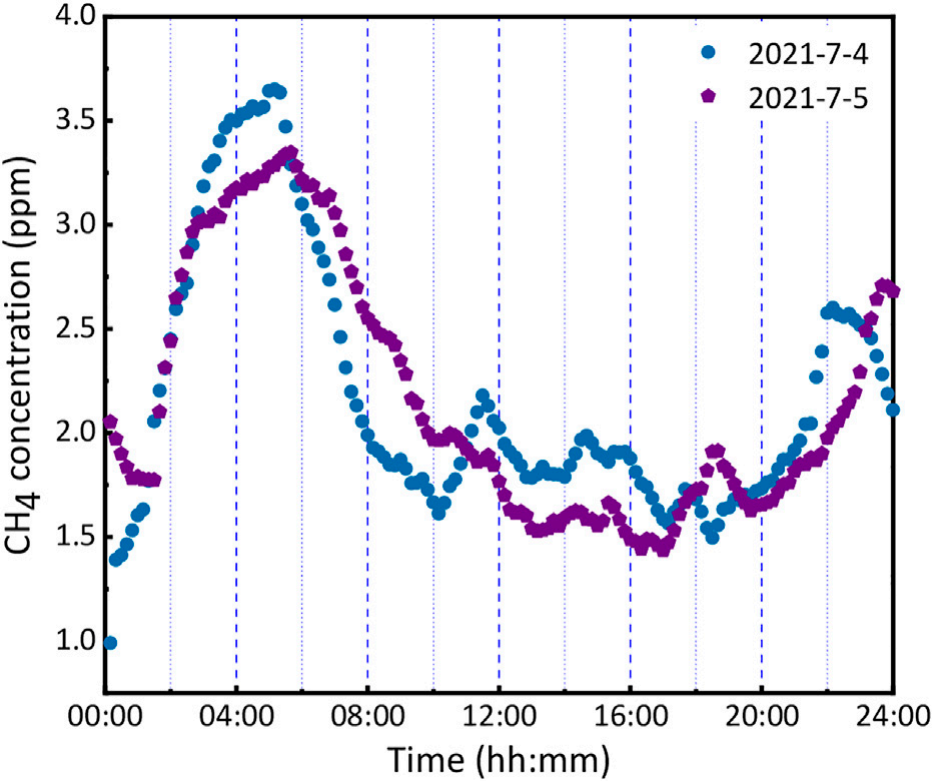
Measured CH_4_ concentrations in atmospheric variation on 2021-7-4(Sunday) and 2021-7-5 (Monday).

The sensor was validated by real-time and *in situ* measurements of atmospheric CH_4_ for 2 days. The test was carried out on the campus of Taiyuan University of Science and Technology, which is a mixed-use area with woods and apartments and is near the Xizhonghuan Express road, which has heavy traffic, especially during rush hours. The weather conditions (19°C33°C and AQI (Air Quality Index) −150) were similar on both days, and complex weather factors, such as thunderstorms and snowstorm, were excluded. In addition, a dryer and a particle filter were installed upstream of the air inlet to suppress the interference of humidity and aerosols. As depicted in Figure 7, the diurnal variation trends of the CH_4_ concentration on these 2 days were the same. The highest concentration of CH_4_ occurred at night, and the lowest concentration occurred during the daytime. The variation in the CH_4_ concentrations during the day was possibly due to the local CH_4_ sources after sunrise (5:12 a.m.), while the concentration increases after sunset (7:55 pm) were probably due to a decreased boundary layer height (Fowler et al., 1996). Moreover, the observed result in Figure 7 clearly shows that more CH_4_ was produced on workdays than on weekends near the road, which explains why vehicle emissions are an important source of CH_4_.

## Conclusion

An enhanced resonant PAS in the near infrared region was developed based on the Herriott type cell for determining the concentration of atmospheric CH_4_. It reaches 34 reflections within the diameter of the beam footprint of approximately 6 mm. The sensitivity of PAS was significantly improved by 13 times in comparison with that of the single pass. The linear relationship between the signal amplitude of the 2nd harmonic and CH_4_ concentration was derived in the region of 200–0 ppm. An Allan variance analysis demonstrated that the MDL accomplished 116 ppb at an integration time of 84 s. The system was deployed for 2 days *in situ* measurement. The results showed that the varying trend in CH_4_ concentration was similar and that of the CH_4_ concentration on working days was slightly higher than that on weekends. The technique is promising for the real-time monitoring of atmospheric CH_4_.

## Data Availability

The original contributions presented in the study are included in the article/Supplementary Material, further inquiries can be directed to the corresponding author.
